# Advancements and Challenges in Self-Healing Hydrogels for Wound Care

**DOI:** 10.3390/gels10040241

**Published:** 2024-04-01

**Authors:** Hossein Omidian, Renae L. Wilson, Erma J. Gill

**Affiliations:** Barry and Judy Silverman College of Pharmacy, Nova Southeastern University, Fort Lauderdale, FL 33328, USA; rw1273@mynsu.nova.edu (R.L.W.); eg1262@mynsu.nova.edu (E.J.G.)

**Keywords:** wound care, self-healing hydrogels, dynamic covalent crosslinking, ionic crosslinking, functional crosslinking

## Abstract

This manuscript explores self-healing hydrogels as innovative solutions for diverse wound management challenges. Addressing antibiotic resistance and tailored wound care, these hydrogels exhibit promising outcomes, including accelerated wound closure and tissue regeneration. Advancements in multifunctional hydrogels with controlled drug release, antimicrobial properties, and real-time wound assessment capabilities signal a significant leap toward patient-centered treatments. However, challenges such as scalability, long-term safety evaluation, and variability in clinical outcomes persist. Future directions emphasize personalized medicine, manufacturing innovation, rigorous evaluation through clinical trials, and interdisciplinary collaboration. This manuscript features the ongoing pursuit of effective, adaptable, and comprehensive wound care solutions to transform medical treatments and improve patient outcomes.

## 1. Introduction

In the complex and evolving field of wound management, healthcare professionals face a range of challenges, particularly with the rise of antibiotic-resistant infections [1]. There is a pressing need for innovative approaches that move beyond traditional antibiotics to reduce the threat of resistance [2]. In this context, self-healing hydrogels have emerged as a significant advancement, offering a design that closely resembles the body’s extracellular matrix. This similarity facilitates a moist environment that is ideal for wound healing [3]. Specifically engineered to include antibacterial properties and the ability to repair wounds, these hydrogels are well-suited for treating chronic wounds [4,5].

The challenge of wound care is further complicated by the need to address specific issues associated with different types of wounds, such as those caused by diabetes or burns. Diabetic wounds, which are characterized by slow healing and a high risk of infection, require careful and tailored management strategies [6,7]. Burns, on the other hand, need dressings that can adapt to the changing nature of the injury, providing both flexibility and strong adhesion [8,9]. The development of self-healing hydrogels that are responsive to changes in pH, sensitive to strain, and capable of promoting wound healing—or those that are infused with therapeutic agents like antioxidants and antibacterial compounds—marks a pivotal shift toward personalized wound care [7,10].

A breakthrough in this field has been the introduction of injectable self-healing hydrogels. These can be applied directly to the wound, offering immediate therapeutic benefits and adapting flawlessly to its shape [11]. This is especially beneficial for treating deep or irregular wounds that conventional dressings cannot properly address [12,13]. However, the development of these hydrogels comes with its own set of challenges. It is crucial to ensure that they are biocompatible and biodegradable to prevent any adverse reactions and allow the body to safely absorb or eliminate them [14]. Additionally, finding the right balance between mechanical strength and the ability to self-heal is essential for hydrogel to withstand the stresses of the wound environment while effectively aiding in the healing process [15,16].

The advancement toward sutureless wound closure has highlighted the need for hydrogel adhesives that can securely attach to tissues and degrade in a controlled manner, minimizing the need for invasive procedures and increasing patient comfort [17]. Furthermore, designing hydrogels that can dynamically self-heal and adapt to changing wound conditions is crucial for sustained and efficient healing [18].

Diabetic wounds, in particular, present a unique challenge, emphasizing the importance of developing dual-drug delivery systems to address various aspects of the healing process [19]. The creation of hydrogels that respond to both pH and glucose levels offers a tailored approach for diabetic foot ulcers, providing smart dressings that adjust to the biochemical changes characteristic of these wounds [20]. Overcoming obstacles such as the rapid degradation of growth factors in diabetic wounds requires innovative strategies to ensure effective delivery of treatments [21].

Recent innovations in hydrogels also focus on sequential drug delivery, utilizing oxidation and reduction reactions for timed release of therapeutic agents, enhancing the healing process [22]. The introduction of luminescent properties in hydrogels opens up new possibilities for real-time monitoring and repair of wounds, improving the accuracy of treatments [23].

Efforts to develop hydrogels with targeted functional crosslinking aim to create materials that not only adhere strongly to tissues but also can be easily removed without causing pain, maintaining their healing efficacy over time [24,25,26,27,28,29,30]. This is particularly important for immediate wound care, where hydrogels with hemostatic properties can significantly impact bleeding control and facilitate healing [30].

The incorporation of metal ion coordination and ionic crosslinking into hydrogel design represents a novel approach to enhancing their antibacterial and adhesive qualities. These innovations focus on effectively addressing local bacterial infections while minimizing the risk of resistance. They achieve this by incorporating multifunctional features into hydrogel dressings, catering to a diverse range of wound care requirements [31,32,33,34,35,36,37,38,39,40,41].

Furthermore, the integration of nanocomposite materials into hydrogels seeks to strengthen their antibacterial and pro-angiogenic capabilities, thus improving their role in promoting skin wound healing [42,43,44,45]. This comprehensive approach highlights the need for adaptable solutions in the face of dynamic wound conditions, where dressings must possess self-healing, bacteria-sensing, and motion-monitoring features to ensure effective and holistic wound care [46,47].

## 2. Self-Healing Hydrogel Polymers

In this review, we explore the significant progress and emerging trends in hydrogel technology, focusing on the selection of polymers, their modifications, and the incorporation of innovative materials and crosslinking methods. The aim is to tailor hydrogel properties for precise applications, with a strong emphasis on biomedical uses.

**Figure 1:**
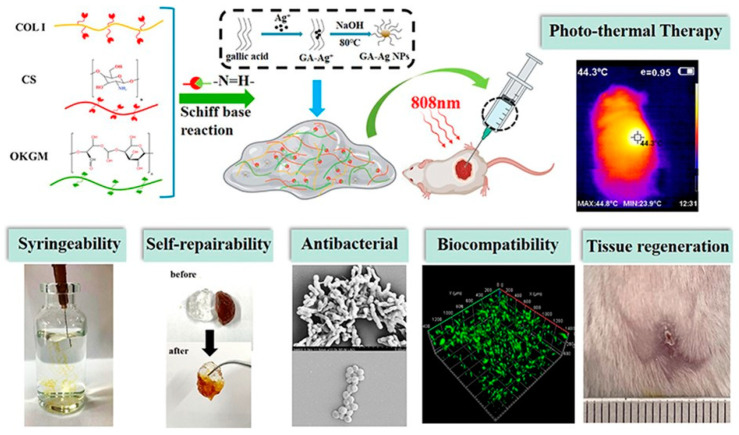
The theory of wound moist healing, an injectable and self-healing hydrogel comprising collagen (COL), chitosan (CS), and oxidation-modified Konjac glucoman-nan (OKGM), which acts as a macromolecular crosslinker to construct dynamic Schiff base bonds [56].

Chitosan stands out as a foundational polymer for numerous hydrogels, appreciated for its versatility in both unmodified and modified forms. Research highlights its broad utility in various fields [2,3,4,6,7,48], with chemical modifications of chitosan [1,5,8,11,14,49,50,51,52,53,54,55] focused on improving aspects like solubility, biocompatibility, and mechanical strength. Combining chitosan with other materials, such as collagen, aims to enhance its biocompatibility and applicability in tissue engineering [10,56]. Notably, the integration of self-healing collagen-based hydrogels with silver nanoparticles, as demonstrated in Figure 1, reveals their potential to provide antibacterial activity and promote tissue regeneration [56].

The industry’s trend toward developing hybrid and composite hydrogels [12,15,52,57,58,59,60,61,62] highlights the pursuit of materials with enhanced properties through the combination of polymers. Collagen’s compatibility with chitosan [10,56] and poly(ethylene glycol) [13] highlights its significance due to its biocompatibility and biodegradability, emphasizing its value in biomedical applications. The exploration into synthetic polymers [63,64,65] reflects a move toward designing materials with specific features such as structural integrity and biodegradability. Similarly, the focus on natural polymers and their modifications [66,67,68,69] indicates a preference for materials that are environmentally friendly and biocompatible.

Innovative crosslinking techniques [9,16,70,71] are being investigated to enhance hydrogels’ physical properties, such as durability, flexibility, and self-healing capabilities. The extensive research into chitosan derivatives [18,19,21,24,29,31,32,33,34,72,73,74,75,76,77,78,79] showcases the ongoing efforts to improve its biomedical applications. Moreover, the exploration of hydrogels featuring novel materials and crosslinking methods [17,23,27,28,30,39,40,41,80,81,82,83,84] reflects a commitment to innovation in developing more effective, responsive, and specialized systems.

The incorporation of alginate, particularly when used alongside dopamine and copolymer chains [85,86], highlights its essential role in drug delivery and tissue engineering, pointing to a focus on biocompatible and responsive hydrogel systems.

This analysis shows clear trends toward enhancing hydrogel functionalities through polymer modification, advanced crosslinking techniques, and the creation of hybrid or composite materials. These developments are part of a broader initiative to customize hydrogel materials for specific purposes, especially for applications such as drug delivery, tissue engineering, and wound healing.

## 3. Crosslinking in Self-Healing Hydrogels

Self-healing hydrogels represent a groundbreaking development in the field of biomedical engineering, particularly in enhancing wound repair methodologies. These materials are ingeniously crafted to emulate the natural healing process of human tissues, possessing a remarkable ability to repair themselves after sustaining damage. This section examines the various crosslinking mechanisms integral to the structure and function of these innovative materials, shedding light on their synthesis and potential uses in medical applications.

At the heart of numerous self-healing hydrogels is the dynamic Schiff base reaction. This process involves the reversible bonding and separation of imine bonds between aldehydes and amines, endowing the hydrogels with the remarkable capacity for self-repair and adaptability to changes in their environment. The reversible nature of Schiff base reactions [3,14,87] enables these materials to autonomously mend themselves. By incorporating modifications such as silver nanoparticles or Fe^3+^ ions [34,37,38,56], these hydrogels not only gain antimicrobial properties but also experience an enhancement in the robustness of their network. Additionally, the incorporation of Schiff bases, along with hydrogen bonds and ionic interactions [12,47,58,77,88,89,90,91,92,93,94,95], serves to amplify their mechanical properties and responsiveness. Enzymatic crosslinking, particularly through the use of horseradish peroxidase [96], fosters the development of complex structures within the hydrogel, significantly enhancing both its strength and its self-healing capacities.

Dynamic imine bonds, including imides and hydrazones, present another avenue for reversible crosslinking, bolstering the hydrogels’ mechanical durability and flexibility, thereby rendering them highly suitable for applications in drug delivery and tissue engineering [2,10,15,57].

Aldehyde-terminated polymers play a pivotal role in forming stable, crosslinked networks within hydrogels. Through covalent interactions with a variety of functional groups, these polymers significantly contribute to the structural integrity and robustness of the hydrogels [1,6,14,50,64,66,67,68].

The application of borate and boronic ester bonds introduces dynamic covalent chemistry to hydrogel networks, enabling self-healing capabilities through reversible bonding. These bonds are instrumental in the development of pH-responsive hydrogels that adeptly adjust to the varying conditions of wound environments [46,63,70,97,98,99,100,101].

Furthermore, the fusion of dynamic covalent and non-covalent bonds, including disulfide linkages and reversible noncovalent interactions, augments the hydrogels’ adaptability and resilience. Certain bonds in this category also introduce redox-responsive features, facilitating the controlled release of therapeutic agents [102,103].

Metal coordination and ionic crosslinking leverage metal ions and polymer interactions to establish networks that are both stable and reversible. This approach enhances the hydrogels’ mechanical attributes and introduces bioactive functionalities, making them particularly effective for wound healing and antibacterial purposes [32,33,35,36,39,104].

Additionally, hydrogen bonding and other non-covalent forces, such as electrostatic and hydrophobic interactions, further refine the hydrogels’ elasticity, strength, and responsiveness. These interactions improve the materials’ mechanical properties and efficiency in self-healing, broadening their applicability in biomedical scenarios [85,105,106].

**Figure 2:**
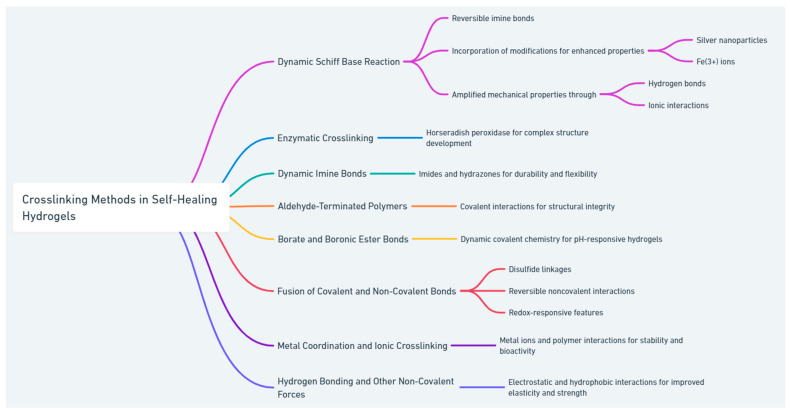
Various crosslinking methods used in the preparation of self-healing hydrogels.

The advancement of self-healing hydrogels through a variety of reversible crosslinking methods marks a significant leap forward in wound repair and tissue engineering. Shown in Figure 2, the diverse crosslinking strategies highlighted here emphasize the material’s versatility and potential, paving the way for their integration into advanced therapeutic and regenerative medical applications.

## 4. Self-Healing Hydrogels Crosslinked via Dynamic Covalent Bonding

### 4.1. Chitosan-Based Hydrogels with Schiff Base and Other Dynamic Linkages

Chitosan-based hydrogels are at the forefront of innovations in wound healing, showcasing a blend of self-repair, antibacterial activity, and biocompatibility in a variety of formulations. One method utilizes neutral glycol chitosan crosslinked with dibenzaldehyde-terminated poly(ethylene glycol) through imine bonds, producing hydrogels with self-healing abilities and inherent antibacterial effects against common pathogens such as *E. coli*, *P. aeruginosa*, and *S. aureus*. This formulation has also shown significant wound contraction capabilities in live models [1]. Another approach involves chitosan-based hydrogels enhanced with quaternized chitosan to leverage its antimicrobial properties. This hydrogel accelerates the healing process in treatments of *S. aureus*-infected wounds without antibiotics, as evidenced in rat skin wound models [2].

Injectable hydrogels represent a notable innovation, with one example being a blend of chitosan and oxidized konjac glucomannan. This mixture uses Schiff base reactions to create adhesive and antibacterial properties, effectively inhibiting *Staphylococcus aureus* and *Escherichia coli* [3]. Another injectable solution combines quaternized chitosan and oxidized pectin, optimized for self-healing, rapid gelation, and mechanical properties appropriate for wound dressings, though it does not exhibit antibacterial properties in extraction media [11]. Moreover, employing dialdehyde bacterial cellulose as a crosslinker in chitosan hydrogels provides injectability, self-healing properties, and antibacterial advantages for wound care. Ascorbic acid enhances the solubility of chitosan in this process [4].

**Figure 3:**
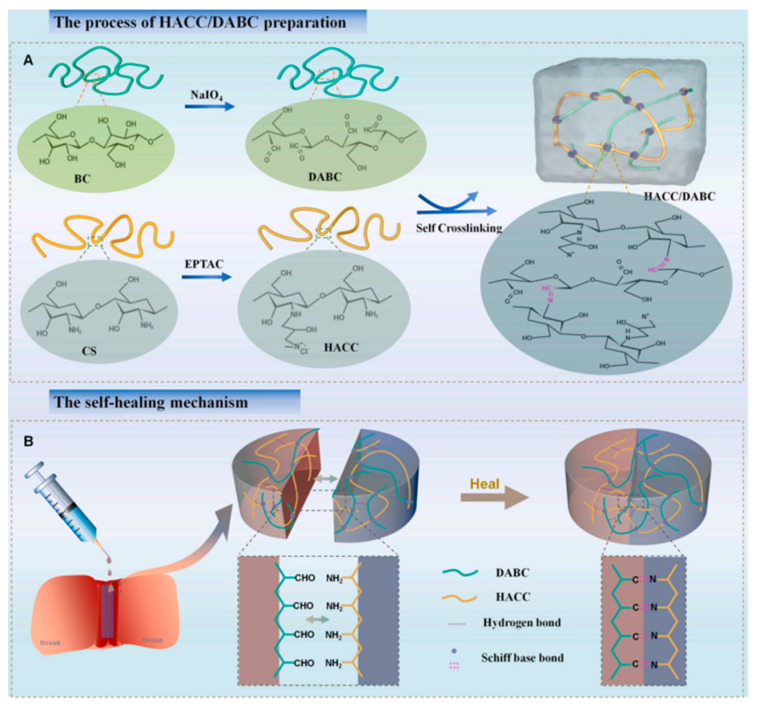
(**A**) The injectable self-healing hydrogel was prepared by Schiff base reaction between amine groups of hydroxypropyltrimethyl ammonium chloride chitosan (HACC) and aldehyde groups of dialdehyde modified bacterial cellulose (DABC). The prepared HACC/DABC hydrogel showed rapid self-healing and injectable properties. (**B**) The multifunctional properties of the hydrogel endow it with huge potential applications in wound healing [5].

Further developments include a hydrogel based on quaternized chitosan and dialdehyde bacterial cellulose, which stands out for its rapid self-healing, injectable application, superior antibacterial activity, and its ability to mimic the natural extracellular matrix (Figure 3) [5].

A novel hydrogel combining collagen, chitosan, and dibenzaldehyde-modified PEG 2000 demonstrates thermal stability, injectability, pH sensitivity, and improved wound healing and hemostatic properties through dynamic imine bonds [10]. An innovative approach features a hydrogel made from chitosan-graft-aniline tetramer and dibenzaldehyde-terminated poly(ethylene glycol), enriched with exosomes for diabetic wound healing, promoting macrophage polarization and angiogenesis [6].

A mussel-inspired chitosan hydrogel, enhanced with lysine and DOPAC, excels in adhesion and rapid self-healing, fostering angiogenesis and better inflammatory responses in wound healing [48]. Furthermore, a composite hydrogel combining collagen, chitosan, and oxidation-modified konjac glucomannan, integrated with silver nanoparticles, shows improved antibacterial activity and wound healing, making it suitable for treating large or irregular wounds due to its syringeability and hemostatic efficiency [56].

Exploring new materials, hydrogels infused with niacin metal-organic frameworks (NOFs), prepared with four-armed benzaldehyde-terminated poly(ethylene glycol) and carboxymethyl chitosan, exhibit antibacterial and antioxidant properties, enhancing wound closure in rats with *E. coli*-infected wounds [14]. Moreover, an injectable hydrogel combining chitosan, hyaluronic acid, and kalium gamma-cyclodextrin metal organic frameworks, loaded with alpha-lipoic acid, has shown promising antibacterial and antioxidant effects [7]. These varied formulations of chitosan-based hydrogels highlight the significant potential of these materials in advancing wound healing technologies, offering tailored properties to address specific clinical needs.

### 4.2. Carboxymethyl Chitosan (CMC) Composites and Hydrogels

Carboxymethyl chitosan (CMC) composites and hydrogels are known for their self-healing properties, biocompatibility, and adaptability to a range of medical needs. A significant advancement in this area is the development of a nanocomposite hydrogel specifically designed for treating deep partial thickness burn wounds. This hydrogel, composed of carboxymethyl chitosan and dialdehyde-modified cellulose nanocrystal, stands out for its ability to dissolve on demand in an amino acid solution, its high capacity for fluid absorption, and its exceptional biocompatibility [8].

Another innovative development is a self-healing, conductive hydrogel wound dressing that incorporates drug and photothermal antibacterial activities. By integrating green-reduced graphene oxide within a carboxymethyl chitosan framework—achieved via Schiff base condensation with oxidized pectin—this hydrogel demonstrates impressive cell viability, electrical conductivity, and enhanced bacteriostatic effects when exposed to near-infrared light [49].

The field has also seen the introduction of an injectable, self-healing carboxymethylated chitosan hydrogel, tailored for wound healing enhanced by mild photothermal stimulation. This hydrogel claims a structure reinforced by dynamic Schiff bonds and incorporates graphene oxide-branched polyethyleneimine. These components enhance its mechanical strength, promote efficient photothermal conversion, and improve wound healing. This translates to benefits such as heightened collagen fiber deposition, enhanced re-epithelialization, and improved formation of granulation tissue [50].

Moreover, a double crosslinked hydrogel combining carboxymethyl chitosan and carboxymethyl cellulose has been created through Schiff base and catechol- Fe^3+^ chelation techniques. This hydrogel is notable for its ability to adapt its shape, superior adhesion, injectable self-healing feature, biodegradability, antibacterial efficacy, and hemostatic properties, making it particularly suitable for dynamic burn wound healing [9]. Additionally, a rapidly self-healing hydrogel has been developed from carboxymethyl chitosan, hydrazide-modified carboxymethyl cellulose nanofibers, and dialdehyde-modified cellulose nanocrystals. The inclusion of nano-reinforced fillers not only enhances the strength of the hydrogel but also ensures its high capacity for liquid absorption [15].

These developments highlight the versatility and potential of carboxymethyl chitosan composites and hydrogels in enhancing wound healing. They represent a new generation of materials that merge user-friendly applications with advanced therapeutic benefits, offering promising solutions for complex wound management.

### 4.3. Specialty Chitosan Derivatives and Crosslinking Methods

Recent progress in synthesizing specialized chitosan derivatives and refining crosslinking techniques has resulted in the development of cutting-edge hydrogels, showing great promise in the fields of wound healing and tissue regeneration. A significant breakthrough is a hydrogel formulated from cysteine-modified carboxymethyl chitosan, sodium oxidized alginate, and but-3-yn-2-one. This hydrogel is crosslinked through Schiff base and thiol-alkynone reactions, exhibiting not only self-healing properties but also antibacterial effects and the promotion of skin tissue regeneration [51].

In addressing diabetic foot ulcers, a self-healing hydrogel comprising N-carboxyethyl chitosan, adipic acid dihydrazide, and hyaluronic acid-aldehyde has been introduced. This hydrogel stands out for its ability to host bone marrow mesenchymal stem cells (BM-MSCs), aiding in regulating the inflammatory response and accelerating the healing process [52]. Likewise, an antibacterial, self-healing hydrogel made from carboxyethyl chitosan and dialdehyde carboxymethyl cellulose, enriched with exosomes from bone marrow mesenchymal stem cells, has proven effective in diabetic wound healing by enhancing angiogenesis and influencing macrophage behavior [53].

For treating Methicillin-resistant *Staphylococcus aureus* (MRSA) infected skin wounds, researchers have developed a hydrogel characterized by its multiple bond crosslinking. This hydrogel distinguishes itself with antibacterial, conductive, and antioxidant properties, coupled with high stretchability and swift self-healing abilities [63]. Another novel approach involves a supramolecular hydrogel based on hydroxypropyl chitosan and poly(N-isopropylacrylamide), crosslinked by beta-cyclodextrin and adamantyl. This hydrogel, loaded with dipotassium glycyrrhizate, offers antibacterial and anti-inflammatory features, making it apt for repairing full-thickness skin wounds [54].

**Figure 4:**
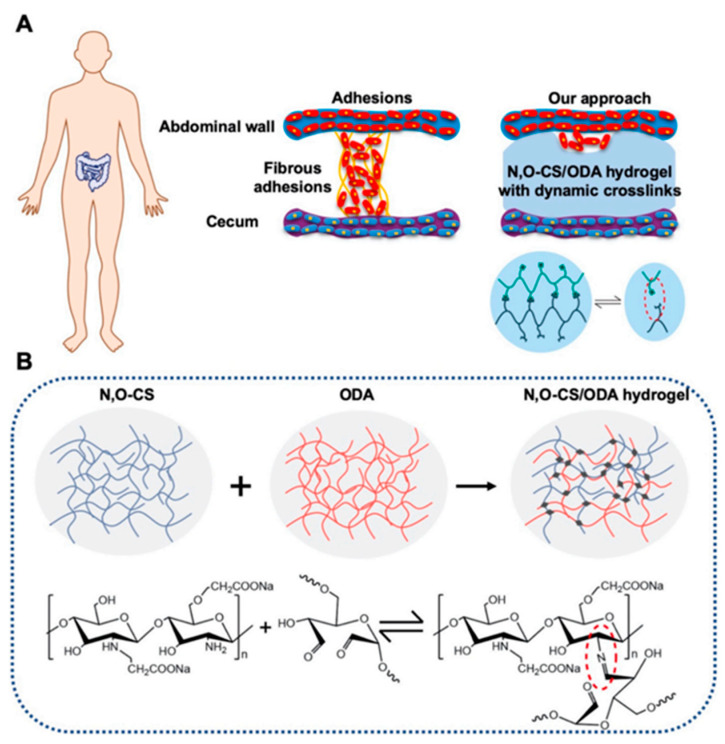
An overview of the novel self-healing, in situ injectable, biodegradable, and non-toxic hydrogels anti-adhesion barrier materials composed of N, O-carboxymethyl chitosan (N, O-CS) and oxidized dextran (ODA). (**A**) Diagram of adhesion formation between two tissues. (**B**) Schematic diagram of covalently crosslinked hydrogels formed by the in-situ polymerization of a precursor macromolecule: by using dynamic crosslinking, shear-thinning, self-healing, and viscoelastic polymer hydrogels that are placed between organs and tissues, these structures are allowed to move freely [55].

Furthermore, an injectable, self-healing hydrogel composed of N, O-carboxymethyl chitosan, and oxidized dextran has been formulated to prevent post-operative peritoneal adhesions. Demonstrating exceptional cytocompatibility and hemocompatibility, as well as antibacterial efficacy, this hydrogel has shown significant potential in reducing peritoneal adhesions in rat models (Figure 4) [55].

### 4.4. Dynamic Crosslinking in Non-Chitosan Polysaccharides and Polymers

Recent research has made significant progress in the dynamic crosslinking of non-chitosan polysaccharides and polymers, leading to the creation of hydrogels with targeted functionalities for wound healing and tissue regeneration. Notably, a poly(aspartic acid) hydrogel, enhanced with hydrazide groups and crosslinked with dialdehyde functionalized poly(ethylene oxide), has emerged. 

This hydrogel includes antibacterial quaternary ammonium moieties and aniline tetramers, enhancing its antibacterial properties and ability to neutralize reactive oxygen species. It has demonstrated significant potential in treating burn wounds due to its exceptional biocompatibility [64].

Another breakthrough is an injectable, self-healing hydrogel crafted from thiol-modified poly(gamma-glutamic acid) and oxidized hyaluronic acid. This hydrogel is designed to emulate the natural extracellular matrix, offering notable biodegradability, free radical scavenging abilities, and enhanced wound healing by fostering angiogenesis and collagen accumulation [66].

Further innovations have been achieved with hydrogels that utilize Schiff base reactions. For instance, a hydrogel synthesized from 1-(propylthio)acetic acid-3-butylimidazole-modified poly(L-lysine) and adipate dihydrazide-modified poly(L-glutamic acid) with oxidized dextran displays injectable and self-healing qualities, along with over 95% effectiveness in eradicating *E. coli* and *S. aureus* [57]. Enhancements in mechanical strength through double-crosslinking have been realized in another hydrogel that combines dynamic Schiff base bonds with photo-induced crosslinking, leading to superior antibacterial effectiveness, tissue adhesion, and wound-healing properties [16].

Mussel-inspired hydrogels, formulated with oxidized konjac glucomannan, gamma-poly(glutamic acid) modified with dopamine and cysteine, and epsilon-polylysine, have been engineered for burn wound treatment. These hydrogels showcase injectable self-healing, antibacterial capabilities, and improved wound healing in burn infection models, characterized by strong adhesion and antioxidant actions [67].

Hydrogels based on aldehyde-carrageenan, integrated with dopamine and zinc ions, present quick recovery features and potent antibacterial activity against *E. coli* and encourage collagen production and fibroblast viability, positioning them as effective for wound healing [58].

Probiotic-laden, injectable, self-healing hydrogels, made from hyaluronate-adipic dihydrazide, aldehyde-terminated Pluronic F127, and fucoidan, are tailored for healing wounds infected by super bacteria. These hydrogels exhibit dose-dependent antibacterial effects and hasten tissue repair [59]. Moreover, injectable adhesive self-healing multi-crosslinked double-network hydrogels, derived from natural polymers, have been developed to facilitate healing in full-thickness skin wounds, offering exceptional injectability, self-repair, and tissue adhesion [12].

In another study, a collagen and poly(ethylene glycol)-based injectable hydrogel, infused with stem cell factor, shows pH-responsive adhesion, rapid self-healing, and enhances diabetic wound repair through stimulating cellular responses and neovascularization [13]. These advancements in dynamic crosslinking for non-chitosan polysaccharides and polymers highlight the cutting-edge potential of hydrogels in medical fields, providing adaptable and effective solutions for improving wound healing and tissue regeneration processes.

### 4.5. Functionalized Polymers and Innovative Crosslinking

A breakthrough has been achieved with the introduction of an injectable adhesive self-healing hydrogel that utilizes a variety of dynamic bonds, such as borate/diol interactions, hydrogen bonding, and Schiff base bonds, enhanced with polydopamine nanoparticles. This design not only facilitates efficient photothermal antibacterial action but also supports the healing of bacteria-infected wounds [70].

Advances in the domain include a self-healing hydrogel synthesized from dopamine combined with pectin hydrazide and oxidized carboxymethyl cellulose, specifically developed for diabetic wound care. This hydrogel capitalizes on mussel-inspired bioadhesion and antioxidation, with capabilities to scavenge reactive oxygen species (ROS), and it effectively delivers epidermal growth factor (EGF), thus accelerating the healing process in diabetic mouse models [60].

Another innovation is a dual glucose/ROS-responsive injectable hydrogel with photothermal antibacterial properties. Incorporating mussel-like catechol groups and phenylboronate ester bond crosslinking and fortified with silver nanoparticles coated with tannic acid (TP@Ag), this hydrogel delivers an impactful anti-inflammatory response and influences macrophage polarization [71].

A multifunctional hydrogel combining bacterial cellulose decorated with polydopamine (PDA@BC) and polyvinyl alcohol through dynamic borate ester bond crosslinking distinguishes itself in treating full-thickness skin wounds. Enriched with doxycycline hydrochloride, it offers superior adhesion, self-healing, and antibacterial activity and promotes efficient wound closure [61].

Cellulose-based self-healing hydrogels, established via boronic ester connections from boronic acid-grafted carboxyethyl cellulose and polyvinyl alcohol crosslinking, cater to both wound healing and antitumor needs. These hydrogels are noted for their excellent biocompatibility, secure adhesion to wounds, controlled release of antitumor drugs, and a reduction in the drugs’ acute toxicity in vivo [62].

A self-healing hydrogel made from hyaluronic acid loaded with Salvianolic Acid B is designed to optimize anti-inflammatory and pro-angiogenesis effects. It employs oxidized hyaluronic acid and adipic dihydrazide-modified hyaluronic acid for its structural integrity [68].

In further contributions to this evolving field, a self-healing hydrogel crosslinked with quaternary ammonium/boronic acid-modified poly(aspartic acid) and polyvinyl alcohol loaded with targeted peptide MP196-conjugated polydopamine has been developed. This hydrogel is engineered for precise antibacterial activity in low pH conditions and photothermal therapy, showing high antibacterial efficiency and rapid healing of infected wounds [65].

In another study, a bionic, self-adhesive hydrogel synthesized from gallic acid-modified epsilon-poly-L-lysine through rapid polymerization stands out for its superior adhesion, mechanical flexibility, and antibacterial, wound-healing, and hemostatic properties [69]. These developments highlight the dynamic potential of crosslinked hydrogels in tackling medical challenges, offering tailored and effective solutions for advanced wound care and related applications.

### 4.6. Composite Materials and Hybrid Hydrogels

One notable advancement is an injectable, self-healing hydrogel adhesive created through a catalyst-free o-phthalaldehyde/amine (hydrazide) crosslinking reaction. These hydrogels excel in providing rapid and durable tissue adhesion, offering controlled degradation within six to twenty-two weeks, ensuring effective hemostasis, and facilitating faster wound healing compared to standard commercial products [17].

Another breakthrough comes from an injectable, self-healing hydrogel intended as a delivery system for fibroblast growth factor 2 (FGF2). Constructed from benzaldehyde-terminated poly(ethylene glycol) (BAPEG) crosslinked with N-Succinyl-chitosan (SCS), it significantly promotes angiogenesis, collagen formation, and granulation tissue development, thereby enhancing tissue repair [18].

Additionally, a self-healing hydrogel comprising N,O-carboxymethyl chitosan-heparin and carboxymethyl cellulose-aldehyde has been designed specifically for diabetic wound healing. It incorporates a dual-drug delivery system for the prolonged release of superoxide dismutase (SOD) and recombinant human epidermal growth factor (rhEGF), improving cellular migration and proliferation and speeding up collagen formation and angiogenesis [19].

The creation of a pH/glucose dual-responsive metformin-release hydrogel for diabetic foot wound healing introduces a multifaceted approach to wound care. This hydrogel is optimized for enhanced adhesion and self-healing capabilities, effectively reducing inflammation and fostering angiogenesis [20].

A self-healing hydrogel made from oxidized microcrystalline cellulose and carboxymethyl chitosan, utilizing a Schiff base reaction, demonstrates pH sensitivity, making it suitable for drug delivery. It also shows strong mechanical integrity, blood clotting efficacy, and controlled rutin release, contributing to improved wound healing [72].

An injectable, self-healing hydrogel developed from sodium alginate and hyaluronic acid with ethylenediamine grafting is tailored for wound dressings. It exhibits covalent crosslinking and controlled release of tetracycline hydrochloride and supports cell migration and wound healing, with its long-term antibacterial effectiveness predicted by one-dimensional partial differential equations [107].

Another self-healing hydrogel, crafted from carboxymethyl chitosan and oxidized hyaluronic acid loaded with taurine, is designed for diabetic wound healing. This hydrogel features pH-responsive and self-healing properties, promotes cell migration, and reduces the production of inflammatory cytokines [73].

An injectable, self-healing hydrogel made from chitosan-tannic acid and oxidized hyaluronic acid is engineered for wound healing. It achieves rapid gelation, biocompatibility, and excellent hemostatic and anti-inflammatory effects and encourages cell growth, significantly expediting the wound healing process in vivo [74].

In another study, a composite hydrogel combining chitosan, silk fibroin, and platelet-rich plasma (CBPGCTS-SF@PRP) has been formulated to safeguard PRP from enzymatic degradation and enhance diabetic wound healing through sustained release [21]. These advancements illuminate the evolving potential of composite materials and hybrid hydrogels in revolutionizing wound care, offering customized, efficient, and forward-thinking solutions for various healing challenges.

### 4.7. Oxidation and Reduction Reactions

In the dynamic arena of biomedical materials research, advancements have also been made in the development of hydrogels that utilize oxidation and reduction reactions, targeted functional crosslinking, and dynamic covalent construction. These advancements aim to optimize drug delivery, wound healing, and tissue repair, featuring controlled release mechanisms and self-healing properties that offer a leap forward in medical treatment efficacy.

A pioneering hydrogel that exemplifies dynamic covalent construction combines aminated gelatin, adipic acid dihydrazide, and oxidized dextran. This hydrogel is engineered for the sequential release of chlorhexidine acetate, an antibacterial agent, and the basic fibroblast growth factor encapsulated within PLGA microspheres. It effectively promotes cell proliferation and wound healing, showcasing the benefits of integrating multiple therapeutic agents for improved health outcomes [22].

Another notable development is a self-healing hydrogel crafted from oxidized carboxymethyl cellulose and PEO dinaphthoate hydrazide, distinguished by its luminescent properties for drug delivery. This hydrogel is celebrated for its superior biocompatibility, its ability to control and sustain drug release, and its potent wound-healing capabilities [23].

### 4.8. Targeted Functional Crosslinking

**Figure 5:**
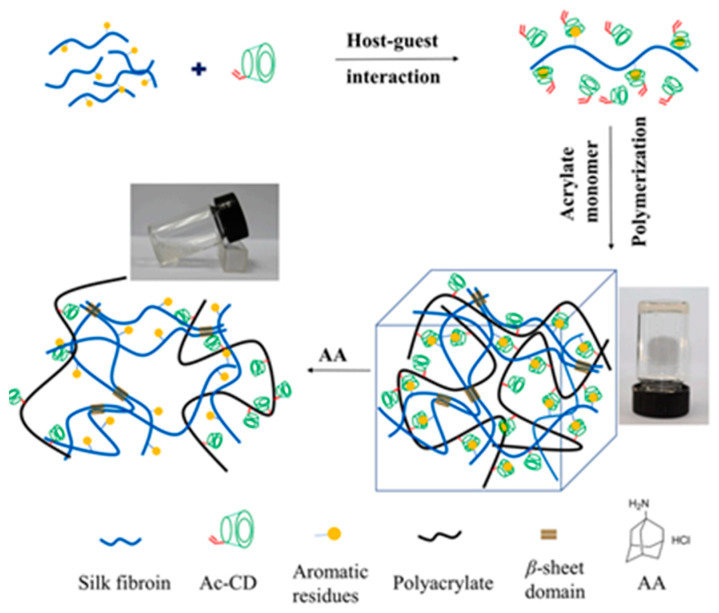
An illustration of a novel self-healing supramolecular Silk Fibroin (SF) based hydrogel with injectability was developed through a simple procedure and fabrication. The precursor of hydrogel was first formed via host–guest interaction between the acryloyl-β-cyclodextrin (Ac-CD) and SF [25].

In targeted functional crosslinking, bioinspired adhesive antibacterial hydrogels have emerged, featuring self-healing and on-demand removability. These hydrogels leverage dynamic host–guest interactions to achieve robust adhesion and swift repair functionality [24]. Silk fibroin-based hydrogels, which incorporate acryloyl-beta-cyclodextrin and 2-hydroxyethyl acrylate, offer rapid self-healing, injectability, and precise drug release control, setting a new standard for medical dressings (Figure 5) [25].

Recent advancements in hydrogel technology have led to the development of ultra-tough, self-healing hydrogels that exhibit cell affinity and tissue adhesiveness, synthesized from dopamine-grafted oxidized sodium alginate and polyacrylamide. These innovative materials showcase remarkable self-healing capabilities, high tensile strength, and exceptional stretchability, highlighting the diverse potential of hydrogel formulations [26].

Further developments have introduced double network hydrogels composed of marine poly- and oligo-saccharides, which are co-enzymatically crosslinked. These hydrogels are notable for their antioxidant and antibacterial properties, toughness, self-healing capabilities, and 3D printability, marking a significant step forward in wound-healing applications [27]. An ultra-tough, self-healable double-network hydrogel, based on salep/polyvinyl alcohol, employs hydrogen bonds and Schiff base crosslinking and is enhanced with Arnebia extract and silver nanoparticles for wound-healing purposes [28].

Additionally, hydrogels crosslinked through multiple dynamic bonds have been developed to accelerate the healing of diabetic wounds by orchestrating the immunoinflammatory microenvironment. These include the integration of cathelicidin LL-37, which contributes antibacterial, immunomodulatory, and neovascularization properties [29]. Hydrogels made from oxidized Bletilla striata polysaccharide and cationic gelatin are designed specifically for skin trauma, offering hemostatic performance and improved wound healing [30]. Hydrogels formulated from oxidized chondroitin sulfate and carboxymethyl chitosan are being utilized as platelet-rich plasma delivery systems for treating diabetic wounds, demonstrating injectability, self-healing, and tissue adhesiveness [75].

Table 1 shows a summary of major polymers used in the preparation of self-healing hydrogels with dynamic crosslinking mechanisms. The table also shows major tests conducted to evaluate the hydrogels for their intended applications.

## 5. Self-Healing Hydrogels with Ionic Crosslinking and Metal Coordination

### 5.1. Metal Ion Coordination and Crosslinking

In advanced wound care, the exploration of metal ion coordination and crosslinking has led to the development of hydrogels that exhibit outstanding self-healing, adhesive, and antibacterial characteristics. These hydrogels aim to enhance wound healing by offering precise delivery of therapeutic agents and by improving the overall effectiveness of treatment approaches.

A significant breakthrough in this area involves the creation of an injectable hydrogel made from carboxymethyl chitosan and oxidized dextran, decorated with carbon quantum dots. This hydrogel, which includes gentamicin sulfate and diammonium citrate for the synthesis of carbon quantum dots, demonstrates self-healing, stretchability, and compressive strengths. Its pH-sensitive release mechanism, combined with effective antibiofilm properties and excellent cytocompatibility, has proven its worth in facilitating wound healing in live models [31].

Hydrogels that are injectable and self-healing, crosslinked with silver and copper ions, have been specifically designed to improve the healing of both infected and diabetic wounds. These hydrogels release silver and copper ions gradually, offering antibacterial and angiogenic benefits, respectively. They are noted for their biocompatibility and suitable adhesive properties [32]. Another development includes an injectable carboxymethyl chitosan hydrogel, integrated with trivalent metal ions like iron and aluminum, showcasing ultrafast gelation and self-healing capabilities. Its adaptability, thermoresponsive nature, and ease of painless removal from the skin promote rapid skin tissue regeneration and wound closure [33].

Antibacterial conductive hydrogels made from oxidized sodium alginate grafted with dopamine/carboxymethyl chitosan and iron(III) exhibit self-healing and near-infrared activated photothermal antibacterial properties, aiding in the healing of infected wounds [34]. Light-responsive wound-dressing hydrogels have been introduced, which strengthen and biodegrade more rapidly under visible light due to the reduction of ferric to ferrous ions [35].

Shear-thinning and self-healing hydrogels, composed of gelatin, vanillin, and iron ions and loaded with andrographolide silver nanoparticles, offer excellent antibacterial activity and promote wound closure in animal studies [36]. Bioinspired hydrogel sealants, comprising sodium alginate, gelatin, protocatechualdehyde, and ferric ions, provide robust bonding strength, injectability, and self-healing and encourage incision healing and closure [37].

Additionally, gelatin hydrogels with enhanced mechanical properties, effective tissue adhesion, self-healing, and antibacterial actions have been developed, facilitated by the interaction between specific phenyl groups and iron ions [38]. Research is also being conducted on injectable, self-healing hydrogels formed by coordinating multi-arm thiolated polyethylene glycol with silver nitrate, exploring their antibacterial and angiogenic effects in the treatment of diabetic skin wounds [39].

Mussel-inspired adhesive and self-healing hydrogels, incorporating sodium dodecyl sulfate surfactant, stearyl methacrylate, N-isopropylacrylamide, dopamine acrylate, and ferric chloride, have been designed for injectable wound dressings. These hydrogels are characterized by their thermo-sensitivity, enhanced mechanical properties, and sustained drug release [40]. Finally, ultra-stretchable, tissue-adhesive, shape-adaptive, self-healing hydrogels are being tailored for healing infected wounds in high-mobility areas, offering strong antioxidative, antibacterial, and hemostasis properties, in addition to photothermal antibacterial capabilities [41]. These advancements highlight the significant potential of hydrogels in biomedical applications, providing innovative and effective solutions for drug delivery, wound care, and tissue repair.

### 5.2. Ionic Crosslinking with Non-Metal Ions

Recent progresses in biomedical engineering have expanded the exploration into ionic crosslinking with non-metal ions, notably within poly(aspartic acid)-based self-healing hydrogels crosslinked with inorganic polyphosphate. These hydrogels exhibit pronounced hemostatic efficiency, antibacterial activity, and tissue regeneration capabilities [80]. Additionally, polyphosphate-conjugated pectin hydrogels, designed for hemostatic and wound-healing applications, demonstrate self-healing properties and effective sustained release [81].

### 5.3. Physical Crosslinking and Self-Assembly

Hydrogel development is also witnessing advancements through physical crosslinking, self-assembly and the utilization of hybrid systems involving ion interactions. These state-of-the-art materials, tailored for a broad spectrum of applications such as treating multidrug-resistant bacterial infections, wound healing, and tissue regeneration, are noted for their rapid shape adaptability, quick self-healing, and stimuli-responsiveness. A notable innovation is the physically double-network hydrogel adhesive, which offers rapid shape adaptability, fast self-healing, antioxidant properties, and sensitivity to near-infrared and pH stimuli, proving particularly efficacious in addressing multidrug-resistant bacterial infections and serving as a removable wound dressing [82]. Another advancement is a methylcellulose-chitosan hydrogel, which addresses hypoxia and infertility in wounds by promoting vascular system reconstruction, thus facilitating the integrated structure necessary for healing [76]. 

### 5.4. Hybrid Crosslinking Systems Involving Ion Interactions

Hybrid crosslinking systems have introduced innovations like MMP-responsive nanoparticle-loaded injectable adhesive self-healing hydrogels, composed of dopamine-functionalized oxidized hyaluronic acid, carboxymethyl chitosan, and collagen. These hydrogels stand out for their injectability, self-healing, tissue adhesion, and biocompatibility, promoting wound healing through controlled curcumin release [77]. Another injectable, self-healing antioxidant hydrogel combines collagen, hyaluronic acid, gallic acid, dopamine, and gamma-poly(glutamic acid) with 3-aminophenylboric acid via dynamic boronic ester bonds, thereby enhancing wound repair [83].

Continuing advancements involve the development of triple-crosslinking conductive self-healing hydrogels, drawing inspiration from mussel adhesion and DNA. These hydrogels integrate calcium ions and sodium alginate to promote wound adhesion, facilitate monitoring, and enable human motion sensing [84]. An injectable self-healing hydrogel featuring copper-tannic acid nanosheets is incorporated into a gelatin-based framework. This formulation provides hemostasis, adhesion, ROS-scavenging, and antibacterial effects and facilitates macrophage polarization and angiogenesis, thereby promoting diabetic wound healing [97].

Additionally, multifunctional chitosan-based hydrogels exhibit self-healing, antibacterial, and immunomodulatory effects, thereby accelerating wound regeneration, reducing bacterial burden, and stimulating angiogenesis [78]. Hydrogels composed of various components enhance near-infrared photo-antibacterial therapeutic effects, improving antibacterial activity and biocompatibility and facilitating infected-wound healing [79].

A photothermal antibacterial antioxidant conductive self-healing hydrogel with nitric oxide release stands out for accelerating diabetic wound healing through efficient photothermal synergistic sterilization and modulation of macrophage M2 polarization [108]. Injectable self-healing hydrogels designed for synergistic treatments showcase photothermal conversion capability and antibacterial activity, advancing melanoma treatment and wound healing [98].

Mussel-inspired antibacterial hydrogels, utilizing aluminum ions, demonstrate mechanical strength, cell affinity, adhesiveness, self-healing, and recycling properties, promoting tissue regeneration [85]. Hydrogels based on polyvinyl alcohol and corn starch offer self-healing properties suitable for wound-dressing applications [99]. A mussel-inspired dual-crosslinking hydrogel made from modified hyaluronic acid and epsilon-polylysine effectively kills bacteria and accelerates wound healing in infected rat wound models [96]. An injectable adhesive self-healing biocompatible hydrogel based on acryloyl-6-aminocaproic acid is designed for hemostasis, wound healing, and postoperative tissue adhesion prevention in nephron-sparing surgery [109].

Table 2 shows a summary of major polymers used in the preparation of self-healing hydropgels with iconic crosslinking and metal coordination. The table also shows major tests conducted to evaluate the hydrogels for their intended applications.

## 6. Self-Healing Hydrogels Using Enhanced Functional Polymers and Responsive Systems

The field of biomedical engineering has made significant progress in developing functional polymers and responsive hydrogel systems for wound care and tissue regeneration. These advancements have introduced materials with conductive, antibacterial, antioxidant, and self-healing properties, vital for treating subcutaneous wounds and various types of wounds, including those affected by methicillin-resistant *Staphylococcus aureus* (MRSA).

Among these innovations are conductive injectable hydrogels tailored for subcutaneous wound-healing applications [110]. Researchers have also engineered quaternized copolymers that form antibacterial hydrogels capable of self-healing and responsive to pH and redox changes, effectively combating bacterial infection and promoting wound healing with improved biocompatibility [102]. Hydrogels designed to respond to temperature changes have shown broad-spectrum antibacterial activity and are particularly effective in treating wounds infected by MRSA, enabling rapid repair [111].

Recent advancements have produced injectable, self-healing hydrogels that eliminate the necessity for chemical crosslinking. These hydrogels demonstrate exceptional cell viability, exhibit effective hemostatic performance in vivo, and possess antibacterial properties [112]. Dual-adhesive hydrogels, loaded with antibacterial agents, deliver potent antibacterial effects against various strains and exhibit good biocompatibility [86]. Zwitterionic hydrogels, which undergo thermal-induced sol-gel transitions, have been optimized for their rapid self-healing capabilities and effective prevention of bacterial adhesion, thereby fostering angiogenesis and speeding up the wound-healing process [113].

Additional innovations include hydrogels designed for increased water absorption and immunomodulatory effects, which have been shown to improve wound repair rates in diabetic rat models [114], and hydrogel/nanofibrous membrane composites that enhance water retention, stretchability, and self-healing capacity, supporting effective therapeutic outcomes in full-thickness skin wound models [88]. Thermoresponsive, self-healing hydrogel dressings, based on triblock copolymers, possess intrinsic antimicrobial properties suitable for treating infected full-thickness wounds [89].

Amphiphilic hydrogels that incorporate exosomes from human umbilical cord blood have been developed to offer enhanced stability and regulated release, featuring temperature-triggered reversible sol-gel conversion [115]. Adhesive hydrogels with anti-inflammatory and antibacterial properties have been shown to accelerate wound healing while minimizing bacterial infection and inflammation [90]. Poly(acrylic acid)-based dressings, known for their strong wet adhesion, aid in fibroblast migration and modulate macrophage polarization to hasten the healing process [116].

**Figure 6:**
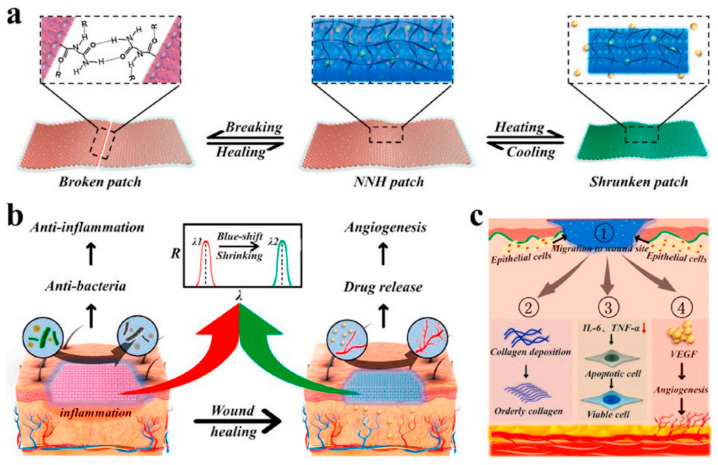
(**a**) The self-healing and temperature-responsive performances of PNIPAM hydrogel (NNH) patches. (**b**) The patches for the treatment of diabetic wounds. (**c**) Schematic illustration of the mechanism of the NNH patch in promoting diabetic wound healing: (1) NNH facilitated the migration of epithelial cells by creating a moist environment. (2) The patch improved the undesirable collagen deposition. (3) The patch controlled wound infection by down-regulating inflammatory factors and (4) promoted the reconstruction of the blood vessels and microcirculation by delivering VEGF to wounds [117].

Supramolecular hydrogels, displaying structural color, mechanical strength, self-healing, antibacterial, and temperature-responsive properties, have been specifically designed for diabetic wound care. An innovative supramolecular hydrogel that changes structural color has been highlighted as particularly suitable for diabetic wound treatment (Figure 6) [117].

FHE hydrogels with stimuli-responsive exosome release have shown promise in enhancing chronic wound healing and skin regeneration [118]. Hydrogels with photothermal and nitric oxide-controlled release have been designed for flexibility, tissue adhesion and accelerated wound healing, alongside biofilm eradication [100].

Recent progress has been achieved in polysaccharide-based hydrogels, which exhibit pH-responsive properties and inherent antibacterial characteristics, coupled with photothermal-assisted bacterial inactivation. These hydrogels are tailored for optimal wound healing, particularly in models with full-thickness skin defects [87]. Additionally, hydrogels incorporating tobramycin and conductive nanowires have been specifically crafted for the healing of burn wounds infected by *Pseudomonas aeruginosa*, showcasing electrical conductivity, antioxidant activity, and the promotion of collagen deposition [91].

Mussel-like hydrogels designed for tendon wound healing exhibit instant self-healing, robust adhesion, and controlled growth factor release [101], presenting a significant advancement in the healing of tendon injuries. Hydrogels that are prepared through photopolymerization under green LED irradiation stand out for their mechanical strength, self-healing capacity, adhesion, and antibacterial properties [119], indicating a novel approach to hydrogel synthesis. Furthermore, injectable hydrogels, crosslinked through dynamic ionic and hydrogen bonds, have been developed to possess antioxidative, antibacterial, and hemostatic activities [105].

Table 3 shows a summary of enhanced functional polymers used in the preparation of self-healing responsive hydrogel systems. The table also shows major tests conducted to evaluate the hydrogels for their intended applications.

## 7. Nanocomposite Self-Healing Hydrogels

Recent developments in biomaterials science have led to the creation of advanced nanocomposite hydrogels, aiming to address the complex requirements of wound healing. These hydrogels, endowed with self-healing capabilities, antibacterial activity, and enhanced biocompatibility, incorporate a variety of nanoparticles and polymers to provide effective solutions for treating infections.

A breakthrough has been achieved with the development of a chitosan/carboxymethyl chitosan/silver nanoparticle composite hydrogel, synthesized through in situ photoreduction. This hydrogel displays potent antibacterial activities and high biocompatibility, proving particularly effective as a wound dressing for wounds infected by *Pseudomonas aeruginosa* [42]. Similarly, injectable, self-healing hydrogels made from antibacterial carbon dots and epsilon-polylysine exhibit a broad spectrum of antibacterial activity, alongside promoting epithelization and angiogenesis, demonstrating exceptional wound-healing efficacy [43].

Advances have also been seen with hydrogels decorated with multifunctional carbon quantum dots derived from herbal medicines, targeting MRSA and offering antibacterial, antioxidative, and anti-inflammatory properties that enhance angiogenesis and wound healing [44]. Hydrogels that incorporate L-arginine conjugated chitosan, functionalized poly(ethylene glycol), and polydopamine nanoparticles also stand out for enhancing angiogenesis and antibacterial activity, thus accelerating wound healing and reducing scar formation [45].

All-hydrogel smart dressing systems featuring gold nanorods, silver nanowires, and dynamic crosslinking strategies have been introduced for real-time wound monitoring, including temperature, strain, and on-demand drug delivery [120]. Injectable self-healing hybrid hydrogels that combine chitosan-based polymers exhibit strong mechanical strength, outstanding self-healing efficiency, and antibacterial properties, promoting healing in diabetic wounds [92].

Exosomes and metformin have been incorporated into self-healing conductive hydrogels with carbon nanotubes to tackle microvascular dysfunction and enhance diabetic wound healing by targeting mitochondrial fission [104]. Ceria-based nanocomposite hydrogels, using cerium oxide nanorods and polyethyleneimine, showcase reactive oxygen species-scavenging activity, indicating significant potential for wound care [93].

Conductive hydrogels integrating hydroxylated graphene bring bioelectronic capabilities to wound dressings, including self-healing, motion monitoring, and bacteria theranostics [46]. Stretchable, self-healing dual dynamic network hydrogels loaded with cuttlefish melanin nanoparticles have been designed for the photothermal clearance of bacteria, effectively treating MRSA-infected motion wounds [47].

pH-responsive hydrogels featuring polyvinyl alcohol, borax, and resveratrol-grafted cellulose nanofibrils have been engineered for bacterial-infected wound management, displaying robust mechanical properties, self-healing efficiency, and excellent adhesion [121]. Antibacterial, antioxidant, electroactive injectable hydrogels that incorporate quaternized chitosan and poly(ethylene glycol) derivatives present self-healing, conductivity, and enhanced wound-healing capabilities [94].

Photoactive self-healing hydrogels using graphitic carbon nitride demonstrate dynamic bonding for self-healing, mechanical strength, tissue adhesiveness, and visible light-induced antibacterial activity, expediting the healing of infected wounds [106]. Hydrogels reinforced with peptide-modified nanofibers through Schiff base dynamic crosslinking improve stability and mechanical strength, offering solutions for chronic wound healing [95].

**Figure 7:**
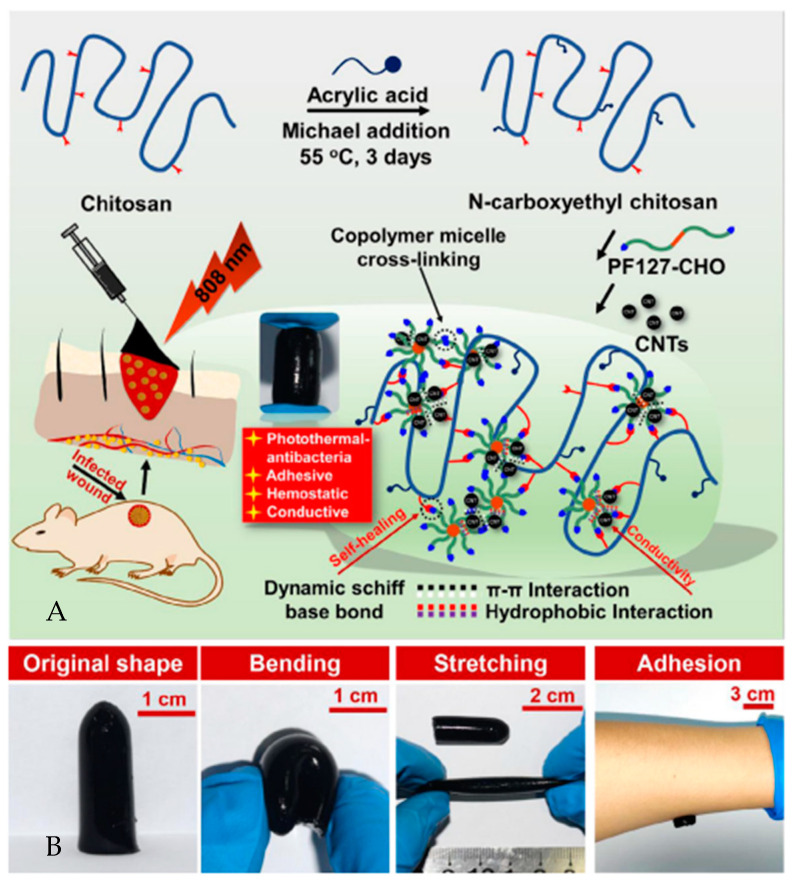
(**A**) A series of conductive self-healing and adhesive nanocomposite hydrogels with a remarkable photothermal antibacterial property based on N-carboxyethyl chitosan (CEC) and benzaldehyde-terminated Pluronic F127/carbon nanotubes (PF127/CNT) were developed, and their great potential as agents for photothermal therapy (PTT) of infected wounds was demonstrated in vivo. (**B**) CEC/PF/CNT hydrogel preparation: original shape, bending, stretching shape representations, and adhesion behavior [122].

Chitosan-graphene oxide hydrogels, aimed at hemostasis and wound healing, feature shear-thinning, self-healing, and adhesiveness [103]. Conductive self-healing nanocomposite hydrogels with photothermal antibacterial properties significantly enhance wound closure, collagen deposition, and angiogenesis, suited for photothermal therapy of infected wounds (Figure 7) [122]. Finally, hydrogels modified with oxidized quaternized guar gum and carboxymethyl chitosan exhibit antibacterial, hemostatic, self-repairing, and injectable properties [123].

Table 4 shows a summary of major polymers used in the preparation of nanocomposite self-healing hydrogels. The table also shows major tests conducted to evaluate the hydrogels for their intended applications.

## 8. Collective Outcomes, Limitations, and Future Directions

### 8.1. Outcomes

The development of materials with innovative functional properties for wound healing marks a significant advancement in the medical field. These materials integrate dynamic Schiff base linkages [1,16,47,70,88,89], incorporate mussel-inspired bioadhesion [60,69], and deploy strategies to enhance hemostasis and tissue adhesion [16,61]. Clinical outcomes have been noteworthy, showing very high wound contraction rates and accelerated healing across various wound types [1,6,8,12,13,16,23,28,32,53,60,73], indicating substantial progress in wound closure, tissue remodeling, and regeneration.

Regarding biocompatibility and safety, there is a pronounced emphasis on ensuring materials’ compatibility with biological systems, as demonstrated by high LC50 values [1] and the utilization of natural, biodegradable [4,30,55,62,72,120,123], and bioactive components [45,95]. Clinical findings have illustrated a reduction in scarring [8,13,24,52] and significant tissue regeneration that emulates the natural extracellular matrix (ECM) [5,25,66,75,82], underlining the critical importance of biocompatibility in medical applications.

Materials possessing antibacterial [3,48,49,56,91,102,112,118,121], antioxidant [14,49,105], angiogenic [6,32,39,45,53], and hemostatic properties [9,10,32,39,41,56,80,97,112] have proven clinically effective in mitigating infection and inflammation [2,14,16,56,57,59,64,67,70] and in fostering angiogenesis and collagen deposition [7,11,18,50,66,83,108,113]. These integrated features are pivotal for biomedical applications, offering a comprehensive healing and tissue repair approach.

Engineered materials for targeted drug delivery [13,20,23,65,68,77,79] with controlled-release mechanisms [19,22,101,104] are in line with clinical advancements, enhancing efficacy. Additionally, materials with advanced functionalities, including photothermal antibacterial activity [10,49,87,122] and real-time monitoring [84,120], play a crucial role in attaining clinical effectiveness.

Hydrogels with broad-spectrum antimicrobial activity and inherent antibacterial properties [102,112,118] align with clinical antibacterial and anti-inflammatory efficacy against various pathogens [1,42,44,91,94,121], showcasing accelerated healing in infection-involved conditions.

Hydrogels featuring self-healing [17,26,87,92], antioxidative activities [105], and targeted drug release mechanisms [18,75,98,108,114] have been effective in managing burns, tendon injuries, and chronic wounds [91,92,101,104], thereby enhancing skin wound healing [45,95,103].

Materials that exhibit superior mechanical properties, such as stretchability [47] and robust tissue adhesion [26,28], have shown clinical superiority over conventional dressings. Their effectiveness in bleeding control [17,69], hemostasis [105,123], and managing internal injuries [17,19,69] makes them adaptable to various wound care scenarios. This highlights the significance of their mechanical properties in diverse clinical applications.

### 8.2. Limitations

The introduction of materials with advanced functionalities into the medical sector presents several challenges, including the complexity of synthesizing these materials, large-scale production, and the associated costs. The intricate processes required for material synthesis are difficult to scale up, leading to significant increases in production costs and complicating mass manufacturing and availability, especially in resource-limited settings [4,9,12,25,33,40,44,67,74,98,111,119]. This difficulty not only impedes the development of such materials but also limits their adoption in clinical practice, representing a significant barrier to their widespread application [3,10,14,53,66].

A lack of comprehensive long-term in vivo studies further complicates the picture. These studies are critical for evaluating new materials’ biocompatibility and safety over extended periods. The current scarcity of data regarding how these materials degrade, the biocompatibility of their degradation products, and their long-term impact on the human body highlights the need for extensive evaluations to ensure the long-term safety and effectiveness of medically intended materials [1,2,5,30,35,42,50,55,56,62,73,76,89,93,99].

Moreover, the effectiveness of these materials and their clinical outcomes varies significantly due to factors such as wound type and location, tissues’ physiological conditions, and individual patient characteristics like pre-existing health conditions [2,11,20,21,34,36,43,58,64,87,88,113]. This variability stresses the need for personalized treatment strategies and further research to tailor applications to individual patient needs.

Finally, navigating the regulatory landscape, addressing ethical considerations, and achieving standardization pose formidable challenges in the innovation and clinical application of new materials [22,65,71,75,77,81,90,114]. The stringent requirements for comprehensive safety and efficacy data, together with the necessity for standardized testing methods and benchmarks, not only delay new materials’ market introduction but also limit their accessibility. These obstacles call for a streamlined approach to overcoming regulatory hurdles, thereby easing the transition from development to clinical usage.

### 8.3. Future Directions

The shift toward developing materials and hydrogel formulations tailored to individual medical needs and patient conditions represents a significant move toward personalized medicine. By customizing these materials [1,5,7,10,13,15,16,18,29,32,41,51,57,63,100,104,108,115], the aim is to improve therapeutic outcomes for various wound types or diseases, integrating smart functionalities for dynamic response and real-time health monitoring. This approach emphasizes the necessity for precision in treatments and the potential to substantially enhance patient care.

Innovation in manufacturing processes is also crucial, addressing scalability, cost-effectiveness, and standardization of therapeutic materials and hydrogels [4,9,12,27,33,40,48,49,52,58,64,67,74,92,98,116,119]. These advancements are designed to make therapeutic aids more accessible and affordable, promoting their broader clinical adoption while ensuring consistent quality. The focus here is on overcoming production challenges to ensure that effective treatments can reach a wider patient base.

Comprehensive evaluations of these materials and formulations through in vivo studies and clinical trials [1,3,6,8,30,35,42,52,55,62,67,73,76,93,99,117] are fundamental. Assessing long-term effects, biodegradability, biocompatibility, and therapeutic efficacy is crucial for optimizing these medical innovations, supporting regulatory approvals, and, ultimately, ensuring their safety and efficacy in real-world applications. This rigorous evaluation process is vital for advancing medical materials from the laboratory to clinical use.

Interdisciplinary collaboration is key to driving these advancements [4,6,24,31,39,45,50,53,56,59,60,69,78,96,118,123]. By bringing together experts from various fields, such as polymer chemistry, material science, biotechnology, medicine, bioengineering, and dermatology, innovative solutions that bridge current gaps in material design, functionality, and clinical application can be developed. This collaborative approach leverages diverse insights and expertise, fostering breakthroughs that could transform medical treatments.

The development of materials and hydrogels with advanced functionalities [2,7,10,54], including controlled drug release, antimicrobial properties, growth factor delivery, and mechanisms for real-time wound assessment, represents a significant advancement. These multifunctional materials offer comprehensive treatment options that adapt to the dynamic needs of wound care, thereby enhancing the healing process. A focus on multifunctionality and advanced features enhances the ongoing pursuit of more effective, responsive, and patient-centered medical treatments.

## 9. Conclusions

The development of innovative materials for wound healing presents remarkable clinical outcomes and promises substantial advancements in medical treatment. These materials, incorporating dynamic linkages, bioadhesion strategies, and multifunctional properties, demonstrate impressive efficacy in wound closure, tissue regeneration, and infection control. However, challenges such as scalability, long-term safety evaluation, variability in clinical outcomes, and regulatory hurdles need to be addressed for widespread adoption. Moving forward, personalized medicine, innovation in manufacturing processes, rigorous evaluation through in vivo studies and clinical trials, interdisciplinary collaboration, and the integration of advanced functionalities are crucial directions. By overcoming these challenges and embracing collaborative efforts, researchers can accelerate the translation of these materials from the laboratory to clinical practice, ultimately revolutionizing wound care and improving patient outcomes.

## Figures and Tables

**Table 1:** Synthesis, testing, and application of self-healing hydrogels prepared using dynamic crosslinking.

Polymer Base and Polymerization Technique	Tests Conducted on Hydrogel	Ref.#
**Chitosan-Based Hydrogels with Schiff Base and Other Dynamic Linkages**		
Neutral glycol chitosan/dibenzaldehyde-terminated poly(ethylene glycol) via imine bonds	High-low strain exposure in continuous step strain rheology; inhibition against *E. coli*, *P. aeruginosa*, *S. aureus*; LC50 values in vivo using zebrafish embryos; wound contraction measurement at 30-dpw	[1]
Quaternized chitosan via imine bonds	Evaluation in a rat skin wound infection model	[2]
Chitosan and oxidized konjac glucomannan via Schiff base reactions	Microscopy and rheological analyses for self-healing, inhibition rates against *Staphylococcus aureus* and *Escherichia coli*, wound recovery time in a full-thickness skin defect model	[3]
Chitosan with dialdehyde bacterial cellulose nanofibers via crosslinking	Not specified beyond being described as injectable, self-healing, with antibacterial properties	[4]
Quaternized chitosan and dialdehyde bacterial cellulose via Schiff base	Rapid self-healing performance, injectable behaviors, superior antibacterial activity, compressive property, water retention, cell proliferation and spreading	[5]
Chitosan-graft-aniline tetramer and dibenzaldehyde-terminated poly(ethylene glycol)	Enhanced diabetic wound healing; promotion of wound healing efficiency; collagen deposition; angiogenesis, M2 macrophage polarization; promotion of HUVEC proliferation, migration, and tube formation	[6]
Chitosan, hyaluronic acid, kalium gamma-cyclodextrin metal organic frameworks loaded with alpha-lipoic acid	Cell proliferation and migration (CCK-8 assay, Transwell experiments), reversal of oxidative stress-induced cell damage, in vivo wound healing in diabetic rats	[7]
Carboxymethyl chitosan and dialdehyde-modified cellulose nanocrystal	Dynamic Schiff base crosslinking, fluid uptake capacity, cytotoxicity assay, three-dimensional cell culture	[8]
Schiff base and catechol-Fe^3+^ chelation double crosslinked hydrogel	Gelation time, mechanical property, adhesive strength, self-healing property, antibacterial activity, hemostatic property	[9]
Collagen, chitosan, and dibenzaldehyde-modified poly(ethylene glycol) (PEG2000) via dynamic imine bonds	Thermal stability, injectability, pH sensitivity, promoting wound-healing and hemostatic ability, strain sensitivity	[10]
Quaternized chitosan/oxidized pectin via Schiff base reactions and ionic interactions	Continuous step strain analysis for self-healing; rapid gelation time; measurements of storage modulus, hardness, compressibility, and adhesiveness; cytotoxicity test on NCTC clone 929 cells; cell migration test; MIC(50) against *E. coli* and *S. aureus*	[11]
Niacin metal-organic frameworks (NOFs) with four-armed benzaldehyde-terminated polyethylene glycol and carboxymethyl chitosan	Self-healing ability through reversible Schiff base reaction, antibacterial and antioxidant activities, in vivo wound closure, tissue formation studies	[14]
Carboxymethyl chitosan, hydrazide-modified carboxymethyl cellulose nanofibers, and cellulose nanocrystals modified by dialdehyde	Fast self-healing, strength reinforcement, liquid absorption capacity	[15]
Chitosan with lysine and 3,4-dihydroxyphenylacetic acid (DOPAC) grafting	Adhesion to the wound surface, promotion of wound healing, antibacterial properties, reduction of inflammatory markers, promotion of angiogenesis	[48]
Collagen, chitosan, and oxidized konjac glucomannan with silver nanoparticles via dynamic Schiff base bonds	Enhanced antibacterial activity, syringeability, self-healing behavior, in vitro and in vivo wound-healing evaluations, hemostatic performance	[56]
**Carboxymethyl Chitosan (CMC) Composites and Hydrogels**		
Carboxymethyl chitosan and oxidized pectin with reduced graphene oxide	Schiff base condensation, cell viability, conductivity, and mechanical strength, photothermal antibacterial activity	[49]
Carboxymethylated chitosan with GO-BPEI (graphene oxide-branched polyethyleneimine) and aldehyde terminated polyethylene glycol	Injectable and self-healing via dynamic Schiff bonds, mechanical strength, photothermal therapy, in vitro cell proliferation, in vivo wound healing	[50]
**Specialty Chitosan Derivatives and Crosslinking Methods**		
Cysteine-modified carboxymethyl chitosan, sodium oxidized alginate, and but-3-yn-2-one based double crosslinking	Scanning electron microscopy, Fourier-transform infrared spectroscopy, rheological testing, antibacterial effect, in vivo biocompatibility	[51]
N-carboxyethyl chitosan, adipic acid dihydrazide, and hyaluronic acid-aldehyde in situ crosslinking	Swelling properties, stability, mechanical properties, regulation of inflammatory environment	[52]
Carboxyethyl chitosan-dialdehyde carboxymethyl cellulose with bone marrow mesenchymal stem cell-derived exosomes	Crosslinking through Schiff base reactions, antibacterial activity, promotion of angiogenesis, transformation of macrophages	[53]
Hydroxypropyl chitosan and poly(N-isopropylacrylamide) via beta-cyclodextrin and adamantyl pre-assembly	Injectable, thermosensitive, highly ductile, self-healable, biocompatible, antimicrobial activity against *Staphylococcus aureus*, in vivo wound healing evaluations on mouse model	[54]
N, O-carboxymethyl chitosan and oxidized dextran without chemical crosslinking agents	Gelation time, cytocompatibility and hemocompatibility, antibacterial activity, biodegradability and biocompatibility, efficacy in preventing post-operative peritoneal adhesion in rat model	[55]
Poly(glycerol sebacate)-co-poly(ethylene glycol)-g-catechol/Zn2+/(3-acrylamidophenyl) boronic acid and 2-hydroxyethyl acrylate/ionic liquids via multiple bonds crosslinking	In vitro antibacterial tests for MRSA and *E. coli*, cyto-compatibility and antioxidation tests, healing effect demonstration on MRSA-infected mouse model	[63]
**Dynamic Crosslinking in Non-Chitosan Polysaccharides and Polymers**		
Dopamine-functionalized oxidized hyaluronic acid, adipic acid dihydrazide-modified hyaluronic acid, and aldehyde-terminated Pluronic F127 via Schiff base dynamic covalent bonds	Injection abilities and mechanical performance testing, self-healing faster than single-network hydrogels, adhesion properties testing	[12]
Collagen and polyethylene glycol with umbilical cord stem cell factor	Rheology, self-shaping and self-healing testing, adhesion strength enhancement, biocompatibility, cellular response, collagen deposition, neovascularization in diabetic rat model	[13]
Dynamic Schiff base bonds and non-dynamic photo-induced crosslinking	Mechanical performance (elastic recovery and tensile modulus), antibacterial capability, tissue adhesion, healing of infectious cutaneous wounds	[16]
Mixed charged polypeptides and oxidized dextran via Schiff base reaction	Injectable and self-healing performances, antibacterial activity tests against *E. coli* and *S. aureus*, hemolysis rate, and cytotoxicity assessment	[57]
Aldehyde-carrageenan with dopamine and zinc ions	Fourier transform infrared spectroscopy (FTIR) analysis, rheological tests, antibacterial properties against *E. coli*, collagen secretion and cell attachment, fibroblast viability	[58]
Hyaluronate-adipic dihydrazide/aldehyde-terminated Pluronic F127/fucoidan with lactobacillus rhamnosus via dynamic Schiff base reaction	Gelation time, mechanical strength, self-healing, and liquid-absorption abilities, anti-super bacteria effect, cytocompatibility and blood compatibility, in vivo wound healing efficacy	[59]
Poly(aspartic acid) with hydrazide functional groups crosslinked with dialdehyde functionalized polyethylene oxide	Antibacterial property, biocompatibility, in vivo burn wound-repairing experiment	[64]
Thiol-modified poly(gamma-glutamic acid) and oxidized hyaluronic acid via thiol-aldehyde addition reaction	Gelation time, rheological behavior, mechanical property, porous structure, degradation process, in vivo wound-healing process in full-thickness skin defect model	[66]
Oxidized konjac glucomannan, gamma-poly(glutamic acid) modified with dopamine and bcysteine, and epsilon-polylysine via thiol-aldehyde addition and Schiff base reactions	Antibacterial effects on *Pseudomonas aeruginosa* and *Staphylococcus aureus*, antioxidant effects, adhesion tests, burn wound infection model healing promotion	[67]
**Functionalized Polymers and Innovative Crosslinking**		
Dopamine coupled pectin hydrazide and oxidized carboxymethyl cellulose for mEGF loading via Schiff base crosslinking	Fast forming and covering of irregular wounds, tissue adhesion for hemostasis, reactive oxygen species scavenging ability, in vivo diabetic wound healing in mice model	[60]
Bacterial cellulose with polydopamine, polyvinyl alcohol via dynamic borate ester bond crosslinking	Tissue adhesiveness, self-healing mechanical property, hemostatic ability, antibacterial activity, swelling ratio, biocompatibility, wound closure in a mouse model	[61]
Carboxyethyl cellulose with boronic acid and polyvinyl alcohol via boronic ester bond crosslinking	Biocompatibility, degradation by cellulase and in vivo, localized injection to cover irregular wounds, drug release carrier for antitumor drugs, in vivo wound repair, and airtight adhesion to the wound site	[62]
Quaternary ammonium/boronic acid modified poly(aspartic acid) and poly (vinyl alcohol) polymers with targeted peptide MP196-conjugated polydopamine	Biocompatibility enhancement, antibacterial efficacy by pH-triggering, photothermal treatment, in vitro synergistic antibacterial efficiency, in vivo healing rate and bacterial survival rate, self-healing property	[65]
Hyaluronic acid with salvianolic acid B via covalent crosslinking of aldehyde groups in oxidized hyaluronic acid and hydrazide groups in adipic dihydrazide-modified hyaluronic acid	Porous structures, self-healing properties, sustainable release capacity, cytocompatibility, full-thickness skin defect model in diabetic rats for wound closure and regeneration	[68]
Gallic acid-modified epsilon-poly-L-lysine and acrylic acid via rapid polymerization under blue light irradiation	Adhesive strength in humid environments, mechanical and self-healing properties, antibacterial ability, wound healing, scar suppression, hemostatic effect on liver bleeding	[69]
Multiple-dynamic-bond crosslinked network of dynamic borate/didiol interactions, hydrogen bonding, and Schiff base bond with polydopamine nanoparticles	Mechanical and adhesive properties, photothermal antibacterial activity, cytocompatibility, and hemocompatibility, in vivo bacteria-infected wound healing	[70]
Glucose/ROS-sensitive dynamic phenylboronester bond crosslinking network with mussel-like super adhesion catechol groups and TP@Ag NPs	Injectable, self-healing, tissue adhesion properties, glucose/ROS-sensitive drug release, photothermal synergistic antibacterial, in vivo infected diabetic wound healing	[71]
**Composite Materials and Hybrid Hydrogels**		
Catalyst-free o-Phthalaldehyde/amine (hydrazide) crosslinking	Rapid and firm adhesion to tissues, controlled degradation profiles in vivo, efficacy in liver and blood vessel injury sealing, rat and rabbit models of full-thickness skin incision healing	[17]
Benzaldehyde-terminated polyethylene glycol and N-succinyl-chitosan via reversible Schiff base reaction	Injectable and self-healing properties, drug encapsulation and implantation, promotion of angiogenesis, collagen deposition, granulation tissue formation	[18]
N, O-carboxymethyl chitosan-heparin and carboxymethyl cellulose-aldehyde via Schiff base crosslinking	Biodegradation, injectable and self-healing properties, drug delivery for moderating microenvironment and promoting cell migration, proliferation, and collagen fiber deposition	[19]
Phenylboronic acid and benzaldehyde bifunctional polyethylene glycol-co-poly(glycerol sebacic acid)/dihydrocaffeic acid and L-arginine cografted chitosan via Schiff base and phenylboronate ester bonding	pH/glucose dual-responsive drug release; adhesion, self-healing, antibacterial, antioxidant, and hemostasis properties; promotion of wound healing in rat diabetic foot model	[20]
Chitosan, silk fibroin, and platelet-rich plasma	PRP protection from enzymatic hydrolysis, sustained PRP release, chemotaxis of mesenchymal stem cells, in vitro proliferation of repair cells, collagen deposition, angiogenesis, and nerve repair in diabetic rat model	[21]
Oxidized microcrystalline cellulose and carboxymethyl chitosan via Schiff base crosslinking	Characterization of hydrogel structures; blood clotting activity; physical properties including gel time, swelling rate, mechanical, and self-healing characteristics; controlled drug release	[72]
Carboxymethyl chitosan and oxidized hyaluronic acid via Schiff base reaction	Physicochemical characterization, biocompatibility, enhancement of cell migration, inhibition of inflammatory cytokines, diabetic rat wound-healing efficacy	[73]
Chitosan modified with tannic acid and oxidized hyaluronic acid via Schiff base reaction	Gel formation process, self-healing capability, biocompatibility, hemostatic performance, anti-inflammatory properties, cell growth promotion, in vivo wound-healing acceleration	[74]
Sodium alginate and hyaluronic acid crosslinked with oxidized sodium alginate	Cell migration, wound-healing promotion, antibacterial property assessment, swelling ratio, biocompatibility, controlled drug release mechanisms	[107]
**Oxidation and Reduction Reactions**		
Aminated gelatin, adipic acid dihydrazide, and oxidized dextran via dynamic covalent reaction	Self-healing capability, antibacterial and sequential drug release, biodegradability, swelling behavior, sequential release of antibacterial agent and growth factor, rat wound-healing effectiveness	[22]
Oxidized carboxymethyl cellulose and polyethylene glycol dinaphthoate hydrazide via acylhydrazone bonding	Gelation time, biocompatibility, in vitro and in vivo drug release study, hemostatic activity, wound-repairing efficacy	[23]
**Targeted Functional Crosslinking**		
Dynamic host-guest interaction-based hydrogel	Strong adhesion property testing, on-demand separation, self-healing properties, protein adsorption properties, mechanical properties, antibacterial properties, biocompatibility, full-thickness rat-skin defect model healing and histomorphological evaluation	[24]
Silk Fibroin, acryloyl-beta-cyclodextrin, and 2-hydroxyethyl acrylate via photo-polymerization	Mechanical properties, long-term stability, rapid self-healing behavior, injectability, biocompatibility, controlled and sustained drug release profile, full-thickness skin defect model healing efficacy	[25]
Dopamine-grafted oxidized sodium alginate and polyacrylamide via hydrogen bonds and dynamic Schiff crosslinking	Self-healing ability, mechanical strength and stretchability, cell affinity and tissue adhesiveness, in vivo and in vitro utility demonstration	[26]
Dual enzymatic co-crosslinking using glucose oxidase/horseradish peroxidase and electrostatic interaction	Toughness, self-healing, moldability, injectability, 3D printability, swelling ratio, biodegradation, antioxidant properties, antibacterial activity, cytotoxicity, cell migration enhancement, blood vessel formation, in vivo wound healing in rat model	[27]
Salep/poly(vinyl alcohol) with ethylene diamine-modified salep and oxidized salep via hydrogen bonds and Schiff base crosslinking	Mechanical strength, tissue adhesiveness, therapeutic properties (cell viability, wound healing), macroscopic evaluation of wound healing	[28]
Multiple-dynamic-bond crosslinked hydrogel with 3,4-dihydroxybenzaldehyde, chitosan, and Fe^3+^ coordinate bonds	Autonomous healing, antibacterial activity, immunomodulation, anti-inflammation, neovascularization, antioxidant activity, diabetic wound-healing promotion	[29]
Oxidized bletilla striata polysaccharide and cationic gelatin via Schiff base reaction	Rheological properties, mechanical behavior, porous structure, degradation performance, hemostatic performance, wound-healing facilitation	[30]
Oxidized chondroitin sulfate and carboxymethyl chitosan with platelet-rich plasma	Gelation, viscoelasticity, inhibition of PRP enzymolysis, sustained growth factor release, cell proliferation and migration, diabetic skin wound-healing acceleration	[75]

**Table 2:** Synthesis, testing, and application of self-healing hydrogels prepared using ionic crosslinking and metal coordination.

Polymer Base and Polymerization Technique	Tests Conducted on Hydrogel	Ref.#
**Metal Ion Coordination and Crosslinking**		
Carboxymethyl chitosan, carbon quantum dots, and oxidized dextran via Schiff base linkage	Self-healing, injectable, stretchable, compressive property, pH-dependent drug release, biofilm inhibition, cytocompatibility, in vivo antibacterial and wound-healing efficacy	[31]
Chitosan crosslinked with silver and copper ions	Antibacterial capability, adhesive ability, water absorption ability, biocompatibility, diabetic wound healing in rat models	[32]
Carboxymethyl chitosan crosslinked with Fe^3+^ and Al(3+) ions	Gelation process, self-healing, self-adaption, thermo-responsive ability, skin tissue regeneration, wound closure	[33]
Oxidized sodium alginate-grafted dopamine/carboxymethyl chitosan/Fe^3+^	Self-healing properties, conductivity, photothermal antibacterial properties, rheological properties, mechanical properties, antioxidant properties, tissue adhesion properties, hemostatic properties, in vivo infected wound healing	[34]
Gelatin and copolymer of dimethyl aminoethyl methacrylate with 2-acrylamido-2-methylpropane sulfonic acid crosslinked with ferric ions	Mechanical strength, pore size via SEM, biodegradation rate under visible light, toxicity (MTT assay for L-929 cell line), histological studies in vivo	[35]
Gelatin/vanillin/Fe^3+^ crosslinked with andrographolide-modified silver nanoparticles	Self-healing capability; swelling degree; antibacterial activity against *E. coli*, *S. aureus*, and *B. pseudomallei*; wound closure in animal models	[36]
Sodium alginate, gelatin, and protocatechualdehyde with ferric ions	Mechanical and adhesive strength, injectability and self-healing capacity, biocompatibility, photothermal antibacterial activity, antioxidation, hemostatic effect, in vivo incision closure evaluation	[37]
Gelatin conjugated with 2-(4’-aldehydephenyl)-4-(2’,3’,4’-trihydroxyphenyl)-2,3-phthalazine-1(2h)-one and Fe^3+^ ions	Mechanical property, tissue adhesion, self-healing capability, biocompatibility, hemostatic and antibacterial activity, wound healing in skin infection rat model	[38]
Multi-Arm thiolated polyethylene glycol crosslinked with silver nitrate	Injectable and self-healing properties, antibacterial and angiogenic in vitro, diabetic skin wound repair, enhancement of angiogenic activity	[39]
Hydrophobic association structure composed of surfactant SDS, stearyl methacrylate, and NIPAAm crosslinked with dopamine acrylate and ferric chloride	Rheological behavior, swelling rate, compression test, adhesiveness on pig skin, self-healing tests, antibacterial tests, in vitro transdermal absorption	[40]
Polyvinyl alcohol, borax, oligomeric procyanidin, and ferric ion	Ultra-stretchability, tissue-adhesive strength, shape adaptability, self-healing feature, on-demand removability, antioxidative, antibacterial, hemostasis, photothermal antibacterial ability, wound healing in mice nape model	[41]
**Ionic Crosslinking with Non-metal Ions**		
Poly(aspartic acid) hydrazide and PEO dialdehyde crosslinked with polyphosphate	Hemostatic performance, biocompatibility, tissue adhesion, in vivo hemostasis model, antibacterial activity, wound repair rate in mouse model	[80]
Pectin conjugated with polyphosphate	Self-healing property, sustained release performance, coagulation characteristic, blood loss reduction in hemorrhage model, wound repair rate acceleration	[81]
**Physical Crosslinking and Self-Assembly**		
Methylcellulose-chitosan hydrogel loaded with exosomes	Cell proliferation, skin remodeling, vascular formation induction, self-healing and adhesion properties, severe wound healing in diabetic conditions	[76]
Double-network hydrogel adhesive based on poly(glycerol sebacate)-co-poly(ethylene glycol)-g-catechol and ureido-pyrimidinone modified gelatin	Shape adaptability, self-healing, tissue adhesion, antibacterial activity, in vivo experiments for wound closure and healing, inflammation regulation, collagen deposition	[82]
**Hybrid Crosslinking Systems Involving Ion Interactions**		
Dopamine-functionalized oxidized hyaluronic acid/carboxymethyl chitosan/collagen hydrogel with curcumin-loaded gelatin nanoparticles	Injectable and self-healing properties, tissue adhesion, biocompatibility, cell proliferation promotion, MMP-responsive curcumin release, animal wound-healing efficiency	[77]
Chitosan and poly[2-(methacryloyloxy)ethyl] trimethyl ammonium chloride	Self-healing ability, biocompatibility, promotion of macrophage M2 phenotype polarization, antibacterial activity, in vivo wound regeneration acceleration	[78]
CuS-grafted-curcumin and carboxymethyl cellulose modified with aldehyde groups and hydroxypropyl trimethyl ammonium chloride chitosan	Injectable and self-healing characteristics, near-infrared photosensitivity, photocatalytic activity, uniform distribution of CuS@C, enhanced photodynamic and photothermal antibacterial effects, accelerated infected-wound healing	[79]
Collagen and hyaluronic acid mediated with gallic acid and dopamine	Injectability and self-healing properties, tissue adhesion, biocompatibility and cell migration, antioxidant and free radical scavenging assays, in vivo wound healing studies	[83]
Calcium ions, sodium alginate, acrylated guanine, acrylamide, and acrylated dopamine	Strong adhesion and peeling properties on various surfaces, high toughness, self-healing property, calcium ions loading for wound monitoring, application in wound bonding on pork stomach	[84]
Aluminum ions, alginate-dopamine, acrylamide, and acrylic acid copolymer	Outstanding mechanical properties, self-healing ability, recycling in pollution-free ways, in vitro antibacterial test, cell affinity, in vivo wound healing	[85]
Hyaluronic acid, epsilon-polylysine, horseradish peroxidase enzymatic crosslinking	Inherent antibacterial properties, gram (+) and (−) bacteria killing, sol-gel transition, recovery from destruction, infected rat wound model efficacy, histological studies comparing with commercial fibrin glue	[96]
Methacrylic anhydride-modified gelatin, cationic guar gum, and borax with copper-tannic acid nanosheets	Hemostasis and adhesion, responsive decomposition for CuTA release, antibacterial efficiency, macrophage polarization and angiogenesis promotion, in vivo re-epithelialization acceleration	[97]
Iron ion-doped polyaniline tethered with guar gum	Injectability, rapid self-healing ability, thermal- or pH-stimuli responsive gel-sol transformations, second near-infrared (NIR-II) responsive photothermal conversion, photothermal-enhanced cytotoxic OH generation, in vitro and in vivo synergistic therapy effects, antibacterial activity, fibroblast cell proliferation, angiogenesis promotion for wound repair	[98]
Poly(vinyl alcohol), corn starch, zeolite, cellulose nanocrystals	Mechanical properties, boric acid and glycerol effects on hydrogel properties, zeolite and cellulose nanocrystals content effects, self-healing tests in wet conditions	[99]
Carboxymethyl chitosan, 2,3,4-trihydroxybenzaldehyde, copper chloride, graphene oxide-N,N’-di-sec-butyl-N,N’-dinitroso-1,4-phenylenediamine	Stability and mechanical properties, conductivity, biocompatibility, photothermal properties and antibacterial activity in vitro, nitric oxide release under NIR, in vivo wound contraction and angiogenesis	[108]
Acryloyl-6-aminocaproic acid (AA) and N-acryloyl 2-glycine (NAG) with calcium chloride (CaCl2)	Mechanical properties, swelling and adhesion properties, flexibility, in vitro blood-clotting ability, cytocompatibility, liver injury and nephron-sparing surgery models for hemostasis performance and wound healing, abdomen-caecum adhesion model for antiadhesion properties	[109]

**Table 3:** Synthesis, testing, and application of enhanced and responsive self-healing hydrogel systems.

Polymer Base and Polymerization Technique	Tests Conducted on Hydrogel	Ref.#
Poly(beta-carboxyethyl acrylate-co-acrylamide) grafted onto sodium alginate loaded with 9-aminoacridine and kanamycin sulfate	Biocompatibility, antibacterial impact on various bacterial strains, rheological properties	[86]
Polysaccharide-based hydrogel with quaternized chitosan (QCS) and oxidized hyaluronic acid (OHA)	Self-healing through reversible Schiff base bonds, NIR irradiation improved antibacterial effect, pH-sensitive drug release, promotion of wound healing in full-thickness skin defect model	[87]
Polyurethane/polydimethylsiloxane nanofibrous membrane and double-crosslinked chitosan-based hydrogel with Schiff base bonds and non-dynamic photo-crosslinking bonds	Water retention capability, stretchable and compressive performance, self-healing and cyclic resilience, in vivo full-thickness skin wound healing	[88]
Poly(N-isopropylacrylamide)-derived ABA triblock copolymer (TNOTN) and aldehyde beta-cyclodextrin (ACD)	Thermal responsiveness and sol-gel transition, self-healing properties, antibacterial properties, biocompatibility and hemocompatibility, in vivo spatial metabolomics for angiogenesis and collagen deposition	[89]
Glycyrrhizic acid (GA)-based hybrid hydrogel with aldehyde-contained GA and carboxymethyl chitosan (CMC)	Injectable, shape adaptation and remodeling, self-healing, antibacterial ability, anti-inflammation effects, in vivo skin wound healing, *S. aureus*-infected skin wound healing	[90]
Quaternized chitosan (QCS), oxidized dextran (OD), tobramycin (TOB), and polydopamine-coated polypyrrole nanowires (PPY@PDA NWs)	Electrical conductivity, antioxidant activity, slow and pH-responsive TOB release, in vitro and in vivo antibacterial activity, NIR irradiation assisted bactericidal activity, PA-infected burn wound inflammation control and healing promotion	[91]
Quaternization chitosan derivatives with phenylboronic acid and catechol-like moieties, BNN6-loaded mesoporous polydopamine (MPDA@BNN6 NPs)	Injectability, flexibility, rapid self-healing, bacterial affinity, tissue adhesion, antioxidant stress ability, in vivo *S. aureus* biofilm eradication, infected wound repair acceleration	[100]
Polyvinyl alcohol (PVA) and hyaluronic acid grafted with phenylboronic acid (BA-HA), polydopamine, and gelatin microspheres containing basic fibroblast growth factor (GMs@bFGF)	Shape-adaptation, strong adhesion, instantaneous self-healing, controlled bFGF release, promotion of tendon wound healing through alleviation of inflammation and collagen I secretion	[101]
Quaternized N-[3-(dimethylamino)propyl] methacrylamide with dithiodipropionic acid dihydrazide	Efficient self-healing capability, pH and redox-triggered gel-sol-gel transition, good antibacterial activity, biocompatibility, degradability, sustained release ability, in vivo experiments for wound closure and cutaneous regeneration	[102]
Quaternary ammonium chitosan (QCS)/tannic acid (TA) hydrogel	Injectability, self-healing, adhesive properties, reactive oxygen species scavenging, broad-spectrum antibacterial ability, rapid hemostatic capability, in vivo hemostasis, wound-healing acceleration	[105]
Poly(ethylene glycol)-co-poly(sorbitol sebacate) conjugated with chitosan-g-poly tetraaniline (QCS-g-PTA)	Antibacterial activity, conductivity, free radical hunt capability, swelling ratio, cytocompatibility, in vivo wound curing development in a full-thickness skin defect rat model	[110]
Itaconic acid-pluronic-itaconic acid (FIA)	Temperature-responsive sol-gel behavior, injectability, broad-spectrum antibacterial activity, hemocompatibility and cell compatibility, intracellular reactive oxygen species scavenging, decrease of inflammation factors, promotion of endotheliocyte migration and blood tube formation, MRSA-infected wound healing, rapid formation of the epithelial layer and skin appendages	[111]
N, O-carboxymethyl chitosan (N, O-CMC) and oxidized chondroitin sulfate (OCS)	Long gelation time and stable performances, cell viability test with NIH/3T3 cells and endothelial cells, antibacterial properties, tissue adhesion, in vivo hemostatic performance	[112]
Poly[(N-isopropyl acrylamide)-co-(butyl acrylate)-co-(sulfobetaine methacrylate)]-b-poly(ethylene glycol)-b-poly[(N-isopropyl acrylamide)-co-(butyl acrylate)-co-(sulfobetaine methacrylate)] (PZOPZ)	Self-healing property, cytocompatibility, antibacterial adhesion, CCK-8, and 2D/3D cell culture experiments, in vivo angiogenesis, dermal collagen synthesis	[113]
Small intestinal submucosa (SIS) hydrogel loaded with Quercetin (QCT)	Self-healing properties, water absorption, immunomodulatory effects, in vivo wound repair rate, promotion of granulation tissue thickness and vascularization, histological analyses of vital organs, biochemical index levels in serum	[114]
ABA-type amphiphilic hydrogel with human umbilical cord blood-derived exosomes (UCB-Exos)	Direct application efficacy, faster wound closure, enhanced collagen deposition, accelerated re-epithelialization, enhanced neo-vascularization	[115]
Polyacrylic acid (PAA) based coacervate hydrogel with isoprenyl oxy poly(ethylene glycol) ether and tannic acid (TA)	Strong wet adhesion; self-healing; extensible properties; antibacterial activity; facilitated fibroblast migration; modulated M1/M2 macrophage polarization; in vivo hemorrhage control; promoted collagen deposition, angiogenesis, and epithelialization	[116]
N-acryloyl glycinamide (NAGA) and 1-vinyl-1,2,4-triazole (VTZ) mixed with supramolecular hydrogel	Superior mechanical properties, self-healing, antibacterial, thermal responsiveness, enhanced wound healing in diabetic rats, color-sensing for wound monitoring	[117]
Injectable, self-healing, and antibacterial polypeptide-based FHE hydrogel (F127/OHA-EPL) with adipose-derived mesenchymal stem cells exosomes (AMSCs-exo)	Materials characterization; antibacterial activity; stimulated cellular behavior; in vivo full-thickness diabetic wound healing; enhanced wound closure rates; fast angiogenesis, re-epithelization, and collagen deposition	[118]
Poly(N-[tris(hydroxymethyl)methyl]acrylamide-co-acrylamide) (P(THMA-co-AAM)) hydrogel with quaternary chitosan	Rapid photopolymerization under green LED, mechanical properties, self-healing, adhesion ability, biocompatibility, antibacterial properties of the tunable multifunctional hydrogel (P-QCS)	[119]

**Table 4:** Synthesis, testing, and application of nanocomposite self-healing hydrogels.

Polymer Base and Polymerization Technique	Tests Conducted on Hydrogel	Ref.#
Chitosan/carboxymethyl chitosan/silver nanoparticles (CTS/CMCTS/AgNPs) polyelectrolyte composite hydrogel	Mechanical strength, self-healing ability, broad-spectrum antibacterial activities, promoting effects on *P. aeruginosa* infected wounds healing	[42]
Antibacterial carbon dots and epsilon-polylysine (CD-Plys)	Broad-spectrum antibacterial activity, wound-healing acceleration, epithelization, enhanced angiogenesis, biocompatibility	[43]
Carbon quantum dots (CQDs) decorated self-healing hydrogel	Antimicrobial against MRSA, ROS scavenging, protection of HUVECs migration and angiogenesis, downregulation of NO and pro-inflammatory cytokines in macrophages, in vivo MRSA elimination, anti-inflammation, promotion of angiogenesis and wound healing	[44]
Polydopamine-functionalized chitosan-arginine hydrogel (CA-pDA) with polydopamine nanoparticles (pDA-NPs)	Self-healing, enhanced mechanical property and pore size, angiogenic and antibacterial activities, acceleration of wound healing with reduced scar formation in rat model	[45]
Polysaccharide biopolymer, poly(vinyl alcohol), and hydroxylated graphene-based conductive hydrogel	Rapid self-healing, injectability, conductivity, motion monitoring, in situ bacterial sensing and killing, in vivo wound-healing promotion, real-time monitoring of joint movements	[46]
Adipic dihydrazide modified hyaluronic acid, benzaldehyde group functionalized poly(ethylene glycol)-co-poly(glycerol sebacate) and cuttlefish melanin nanoparticles	Tissue adhesion, stretchability, self-healing, photothermal antibacterial therapy, antioxidation, hemostasis, exudate absorption, sustained release property, in vivo wound healing	[47]
Chitosan-based POSS-PEG hybrid hydrogel via Schiff base reaction between HACC and POSS-PEG-CHO	Mechanical strength, injectability, self-healing efficiency, cytocompatibility, antibacterial properties, in vivo diabetic wound-healing acceleration	[92]
Ceria-based nanocomposite hydrogel (FVEC) via dynamic Schiff base reaction	Thermosensitivity, injectability, self-healing ability, ROS scavenging activity, in vivo biocompatibility and biodegradability, full-thickness skin wound healing enhancement	[93]
Quaternized chitosan-g-polyaniline and benzaldehyde group functionalized poly(ethylene glycol)-co-poly(glycerol sebacate)	Self-healing, electroactivity, free radical scavenging, antibacterial activity, adhesiveness, conductivity, swelling ratio, biocompatibility, in vivo blood clotting capacity, wound-healing process enhancement	[94]
Peptide-modified nanofibers and hydrogel via Schiff base dynamic crosslinking	Stability, mechanical strength, injectable, self-healing, antibacterial, hemostatic properties, chronic wound-healing acceleration	[95]
Chitosan and graphene oxide via one-pot heating	Injectability, self-healing, mechanical and rheological properties control, adhesiveness, hemocompatibility, in vivo hemostatic and wound-healing capability	[103]
Exosome/metformin-loaded PEG/Ag/CNT hydrogel with dynamic Schiff base bonds	Tissue adhesiveness, antioxidant, self-healing, electrical conductivity, cell proliferation and angiogenesis promotion, peritraumatic inflammation and vascular injury relief, ROS reduction, mitochondrial fission interference	[104]
Carboxymethyl chitosan, tannic acid, and graphitic carbon nitride	Self-healing capability, mechanical property, tissue adhesiveness, cell affinity, visible light-induced antibacterial activity, collagen synthesis and re-epithelization acceleration, bacteria-infected wound healing	[106]
PNAGA/AuNRs, PNAGA/PNIPAm, and PNAGA/AgNW hydrogels integrated by self-healing crosslinking	In vitro antibacterial experiments, in situ rat models for wound monitoring and healing, real-time monitoring of wound temperature and strain, on-demand drug delivery	[120]
Poly(vinyl alcohol)-borax and resveratrol grafted cellulose nanofibrils	Mechanical properties, self-healing efficiency, adhesion performance, pH-responsive drug release, biocompatibility, antioxidant effect, in vitro and in vivo antibacterial, skin tissue regeneration, wound closure capabilities	[121]
N-carboxyethyl chitosan (CEC) and benzaldehyde-terminated Pluronic F127/carbon nanotubes (PF127/CNT)	Gelation time, mechanical properties, hemostatic properties, water absorbency, biodegradability, pH-responsive antibiotic release, antibacterial activity, photothermal antimicrobial activity, conductivity, in vivo healing in infected wounds	[122]
Oxidized quaternized guar gum (OQGG) and carboxymethyl chitosan (CMCS)	Structural characterization, antibacterial, hemostatic, self-repairing, injectable properties, cytocompatibility, in vivo wound-healing promotion in an *S. aureus*-infected rat model	[123]

## Data Availability

Not applicable.
